# Insights into Tissue-specific Specialized Metabolism in *Tieguanyin* Tea Cultivar by Untargeted Metabolomics

**DOI:** 10.3390/molecules23071817

**Published:** 2018-07-21

**Authors:** Si Chen, Jun Lin, Huihui Liu, Zhihong Gong, Xiaxia Wang, Meihong Li, Asaph Aharoni, Zhenbiao Yang, Xiaomin Yu

**Affiliations:** 1 College of Horticulture, Fujian Agriculture and Forestry University, Fuzhou 350002, China; cstc1990@hotmail.com (S.C.); liuhh42194@foxmail.com (H.L.); limei123home@gmail.com (M.L.); 2 FAFU-UCR Joint Center for Horticultural Biology and Metabolomics, Fujian Provincial Key Laboratory of Haixia Applied Plant Systems Biology, Fujian Agriculture and Forestry University, Fuzhou 350002, China; realnadal@163.com (J.L.); zhihong_gong@sina.com (Z.G.); wangxiaxia530@126.com (X.W.); yang@ucr.edu (Z.Y.); 3 Department of Plant & Environmental Sciences, Weizmann Institute of Science, P. O. Box 26, Rehovot 7610001, Israel; asaph.aharoni@weizmann.ac.il; 4 Center for Plant Cell Biology, Institute for Integrative Genome Biology, and Department of Botany and Plant Sciences, University of California, Riverside, CA 92521, USA

**Keywords:** oolong tea, *Tieguanyin* tea cultivar, metabolite profiling, UPLC-QTOF MS, metabolomics

## Abstract

Tea plants produce extremely diverse and abundant specialized metabolites, the types and levels of which are developmentally and environmentally regulated. However, little is known about how developmental cues affect the synthesis of many of these molecules. In this study, we conducted a comparative profiling of specialized metabolites from six different tissues in a premium oolong tea cultivar, *Tieguanyin*, which is gaining worldwide popularity due to its uniquely rich flavors and health benefits. UPLC-QTOF MS combined with multivariate analyses tentatively identified 68 metabolites belonging to 11 metabolite classes, which exhibited sharp variations among tissues. Several metabolite classes, such as flavonoids, alkaloids, and hydroxycinnamic acid amides were detected predominantly in certain plant tissues. In particular, tricoumaroyl spermidine and dicoumaroyl putrescine were discovered as unique tea flower metabolites. This study offers novel insights into tissue-specific specialized metabolism in *Tieguanyin*, which provides a good reference point to explore gene-metabolite relationships in this cultivar.

## 1. Introduction

Tea is the world’s most consumed beverage, second only to water. The popularity of tea can be partly accounted for by the diversity of its taste and aroma, owing to the diversity and abundance of specialized metabolites in tea. Tea consumption has also been linked to a number of medicinal and nutritional properties resulting from a wide array of phytochemicals in tea plants (*Camellia sinensis*) [1]. Great efforts have been made by the tea research community to functionally characterize the bioactive components in tea. In particular, catechins, caffeine, and theanine, three of the most characteristic metabolites known to be closely associated with tea flavor and quality, have been extensively studied molecularly and biochemically [2,3,4,5,6,7,8]. With the recent release of genome sequences for *C. sinensis* var. *sinensis* [9] and *C. sinensis* var. *assamica* [7], new insights into the molecular basis for the rich production of bioactive metabolites in tea plants will likely emerge.

The production of specialized metabolites is believed to be employed by plants mostly for the purpose of chemical defense or communication, increasing the overall fitness of the given plant producing them [10]. As with many other plants, specialized metabolism in tea plants varies in a tissue and species-specific manner, and is sensitive to both biotic and abiotic cues [7,11]. The biosynthesis of catechins, caffeine, and theanine in response to developmental cues has been most studied in tea plants [7,9]. Catechins are derived from the phenylpropanoid and flavonoid pathways [12]. The biosynthesis of caffeine involves three methylation steps to sequentially convert xanthosine to 7-methylxanthine to theobromine, and then finally to caffeine [13]. Theanine biosynthesis is catalyzed by theanine synthase acting on glutamate and ethylamine as substrates [14]. By RNA sequencing of various tissues from different developmental stages of cultivar *Longjing 43*, Li et al. analyzed the expression patterns of genes involved in the biosynthesis of flavonoids, caffeine, and theanine, and built a possible transcription factor network for the regulation of these three pathways [12]. Developmental changes in the abundance of catechins [3,11], caffeine [5,15], and theanine [5,6], and the differential expression of relevant genes in respective pathways were also documented in other tea cultivars. Nevertheless, most studies have largely focused on one or several classes of target metabolites, questions about how gene expression affects the metabolic make-up, and the distribution patterns of specialized metabolites in different tissues are not fully understood. As transcriptomics alone could not reflect the actual biochemical status (and hence the real physiology) of tea plants, non-targeted metabolomics, which involve the qualitative and semi-quantitative detection of a high number of metabolites participating in various cellular activities, is required for a more direct and comprehensive measurement of biological activities in the individual tissues of tea plants. The same approach has been successfully applied to profile many plant species during development or in response to changing environmental stress [16,17,18,19]. However, the application of untargeted metabolomics to examine the overall difference in the metabolic profiles among tea plant tissues has not been thoroughly performed in any tea cultivars.

Based on the methods of tea leaf processing, tea has been categorized into six major types: green, yellow, oolong, white, black, and dark tea [20]. *Tieguanyin* tea, originating from Anxi County, Fujian province of China, is a premium variety of oolong tea renowned for its uniquely rich flavors and various health benefits [21,22,23]. Due to its increasing popularity among consumers, the plantation of *Tieguanyin* tea cultivar has been greatly expanded, spreading from Anxi and surrounding areas in the Fujian province to many other regions in China. A thorough understanding of the biology and metabolism of *Tieguanyin* tea plants would facilitate the development of high-quality tea products but remains underexplored. To date, a limited number of available studies on this cultivar have only focused on the geographic origin discrimination and targeted analyses of chemical changes during processing using processed tea [7,21,24,25].

In the present study, we comprehensively investigated the phytochemical profile of *Tieguanyin* cultivar by applying a non-targeted metabolomics workflow, with the aim of revealing the differences and similarities in the metabolite composition among different tissues and identifying the tissue-specific distribution patterns of specialized metabolites. Our results provide novel insights into the developmental regulation of the specialized metabolism in *Tieguanyin* and reveal intriguing variations in the diverse classes of metabolites besides known compounds. It likely offers a valuable reference for future characterizations of the gene–metabolite relationships of metabolites uncovered in the current study.

## 2. Results

### 2.1. Prominent Metabolite Variations Observed between Tea Plant Tissues

**Figure 1:**
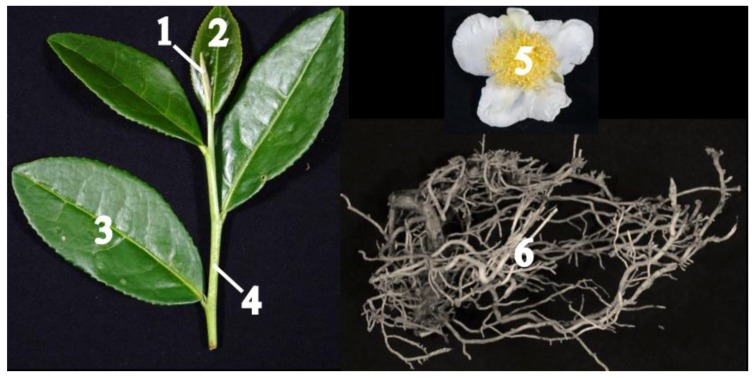
Phenotypic characterization of six tissues of *Tieguanyin* tea plants used in the current study. (1) bud, (2) young leaf, (3) mature leaf, (4) new stem, (5) flower, and (6) lateral root.

**Figure 2:**
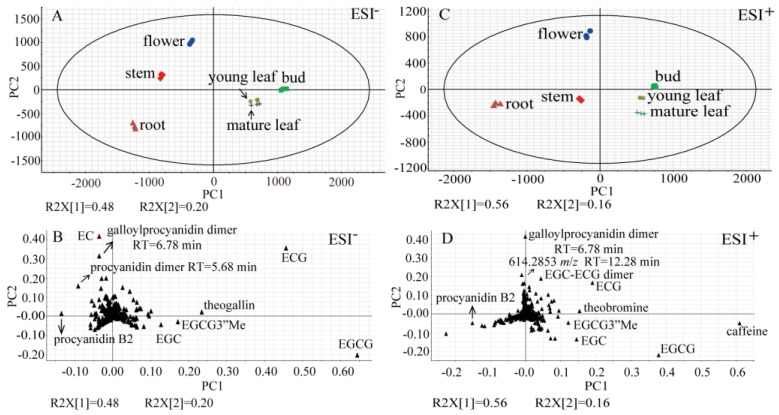
Metabolic profiles of tea tissue samples demonstrated by PCA score plots and PCA loading plots. (**A**) PCA score plot for tea tissue samples based on 732 single molecular features detected in ESI^−^. (**B**) PCA loading plot showing major metabolites that contribute to group separation in ESI^−^. (**C**) PCA score plot for tea tissue samples based on 821 single molecular features detected in ESI^+^. (**D**) PCA loading plot showing major metabolites that contribute to group separation in ESI^+^. R2X, explained variation. PC1, the first principal component. PC2, the second principal component. For each tissue type, three biological replicates were prepared, where one replicate was a pool of collected materials from three tea plants.

**Figure 3:**
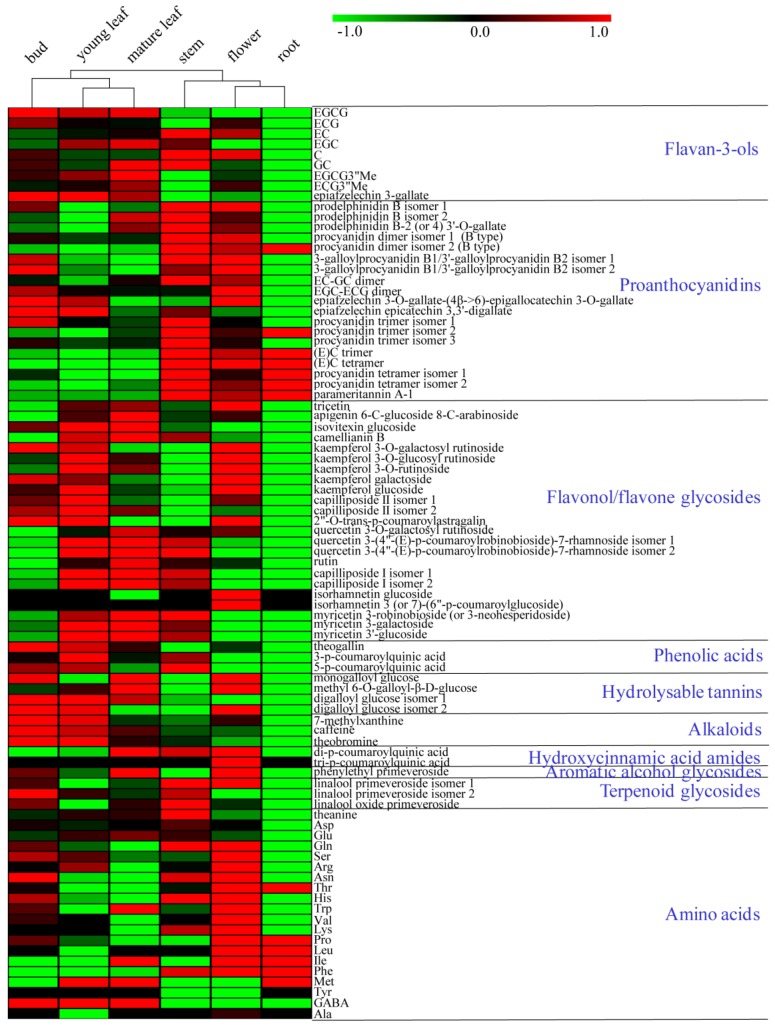
Comparisons of metabolite levels in six tissues. The analysis is based on the normalized average signal abundance from three biological replicates for each tissue type. Normalized values are shown on a color scale proportional to the content of each metabolite, and are expressed as log2 using the MultiExperiment Viewer software (MeV v4.9.0, J. Craig Venter Institute, La Jolla, CA, USA).

To assess metabolite compositional differences between tissues of *Tieguanyin* tea plants, non-targeted analysis based on UPLC-QTOF MS (ultra-performance liquid chromatography-quadrupole time-of-flight mass spectrometry) was carried out to profile methanol-soluble extracts of buds, young leaves, mature leaves, new stems, flowers, and lateral roots (Figure 1 and Figure S1). Metabolite profiles were presented as PCA score plots, PCA loading plots, and the heat map (Figure 2 and Figure 3). A total of 68 differential compounds (VIP > 1 and *p* < 0.05) were tentatively identified on the basis of their accurate masses, MS/MS fragmentation patterns, and UV absorbance, in comparison to standard compounds and references (Table 1). They were classified into 11 major classes including flavan-3-ols, proanthocyanidins, flavonol glycosides, flavone glycosides, phenolic acids, hydrolysable tannins, alkaloids, hydroxycinnamic acid amides, amino acids, aromatic alcohol glycosides, and terpenoid glycosides.

In the PCA score plot in ESI^−^ (electrospray ionization in the negative ion mode), the first principle component (PC1) and the second principal component (PC2) explained 48.0% and 20.0% of the variation, respectively (Figure 2A). Except for leaves from different developmental stages that were clustered, the remaining samples were clearly separated from each other at both of the PC1 and PC2 axis, suggesting distinct metabolic profiles among the tea tissues. To further depict major differential metabolites, the PCA loading plot was applied. Along PC1, (–)-epigallocatechin gallate (EGCG), (−)-epigallocatechin 3-(3-*O*-methylgallate) (EGCG3”Me), theogallin, and procyanidin B2 were observed as the main contributors toward the discrimination of buds and leaves from other tissues (Figure 2B). Along PC2, (−)-epicatechin (EC), one galloyl procyanidin dimer, and a second procyanidin dimer were responsible for the separation of stems and flowers with the remaining tissues. The separation pattern observed in the PCA score plot in ESI^+^ was similar to that in ESI^−^, where PC1 was 56.0% and PC2 was 16.0% (Figure 2C). Buds and leaves were grouped together, while the remaining samples were separated at the PC1 axis. Flowers were segregated from other tissues at the PC2 axis. In addition to the compounds observed in ESI^−^, caffeine and theobromine contributed significantly (*p* < 0.05) to the separation of buds and leaves from other tissues. An EGC-ECG dimer and an unknown metabolite (*m*/*z* = 614.2853, RT = 12.28 min) were found to occur more abundantly (*p* < 0.05) in flowers (Figure 2D).

### 2.2. Structural Compositions of Oligomeric Proanthocyanidins Varied by Tissue Types

Proanthocyanidins (PAs) are a group of structurally complex oligomeric (degrees of polymerization or DP = 2–10) or polymeric (DP > 10) flavan-3-ols linked by interflavan C-C bonds. PAs are remarkably diverse as a result of the diversity of monomeric units, types of linkages, and variations in chain lengths [30]. Authentic standards for most PAs are not commercially available. Moreover, reports on the purification, identification, and distribution of PAs in tea plants are limited [29,30]. Therefore, unambiguous structural assignments for PAs are quite challenging. Nonetheless, according to the MS/MS fragmentation patterns previously described [41], we tentatively identified **19** oligomeric PAs by UPLC-QTOF MS, among which 10 were procyanidins (compounds **14**–**20**, **22**, **23**, **25**), two were galloylated procyanidins (compounds **24** and **28**), two were prodelphinidins (compounds **10** and **11**), one was a galloylated prodelphinidin (compound **13**), two were procyanidin/prodelphinidin dimers (compounds **12** and **21**), and two were propelargonidins (compounds **26** and **27**) (Table 1, Figure S2). PAs with DPs higher than four were outside of our detection window (50–1200 Da), and hence not included in the analysis. B-type PAs, which are characteristic of C4→8 or C4→6 interflavan bonds, were predominant in tea plants and were found to exist as dimers, trimers, and tetramers. Several isomers of procyanidin oligomers were observed to elute at different times. For example, compounds **14**, **15**, and **20** were all assigned as B-type procyanidin trimers (Figure S3), and compounds **18**, **19**, and **23** were all assigned as B-type procyanidin tetramers (Figure S4).

**Figure 4:**
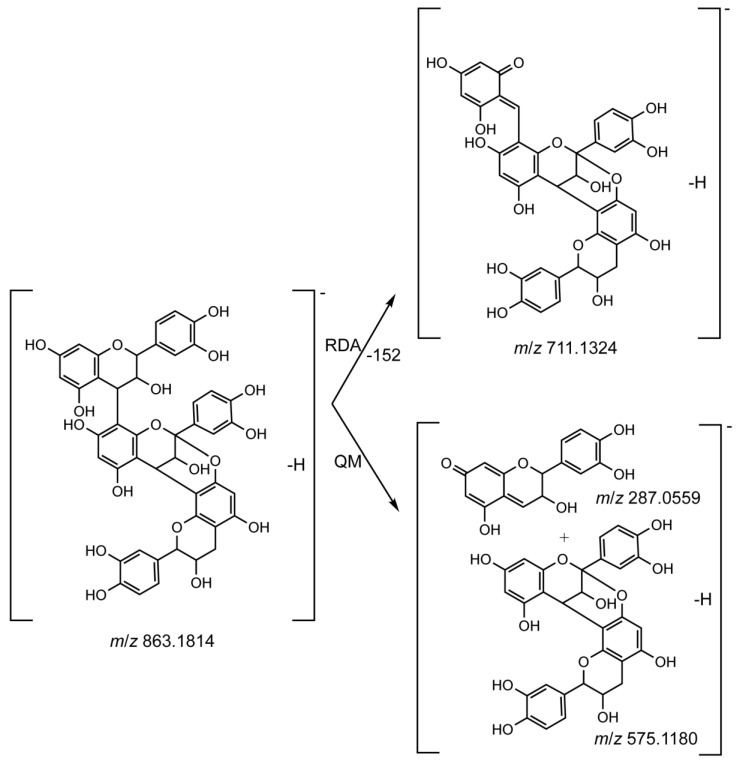
Proposed fragmentation pathways for compound **22**, a possible A-type procyanidin trimer, based on generated fragment ions.

In addition, two less common A-type PAs, which were characterized with an additional ether linkage between C2→7, were detected in roots and stems. A-type PAs are readily recognizable because their *m*/*z* values are two Da less than corresponding B-type PAs [41]. Compound **22**, with *m*/*z* 863.1814 in ESI^−^, was two Da lower in mass than B-type procyanidin trimers. The fragmentation of compound **22** yielded a fragment at *m*/*z* 711.1324, as a result of retro Diels-Alder (RDA) cleavage (Figure 4 and Figure S5). Subsequent water elimination generated a fragment at *m*/*z* 693.1232. Other key fragments such as *m*/*z* 575.1180 and 287.0559 were derived from quinone methide (QM) fission (Figure 4 and Figure S5). As a result, compound **22** was speculated as (E)C-(4→8)-(E)C-(2→7, 4→8)-(E)C. Similarly, the protonated ion of compound **25**, with formula C_60_H_48_O_24_, had *m*/*z* at 1153.2572. This was two Da less compared with B-type procyanidin tetramers, which is suggestive of a tetrameric PA containing one additional A-type linkage. Low signal intensity made it difficult to compare the spectrum with known compounds. Nevertheless, the fragment ion we observed at *m*/*z* 1001.2155 (Table 1) may arise from the RDA cleavage. One compound described previously in the barks of other plant species had the same formula and was identified as parameritannin A1, namely, EC-(2*β*→O7, 4*β*→8)-[EC-(4*β*→6)]-EC-(4*β*→8)-EC [32,33]. To the best of our knowledge, this is the first description of these two A-type PAs in tea plants.

Semi-quantitative comparisons of PAs and monomeric catechin units among tissues revealed that the DP of PAs increased from the upper part of tea plants to the lower part (Figure 3 and Figure S2), which is in line with the results from other studies [11,30]. The upper part of tea plants, particularly buds and leaves, was rich in monomeric catechins, including EGCG, (−)-epicatechin gallate (ECG), EC, (−)-epigallocatechin (EGC), (+)-catechin (C), (−)-gallocatechin (GC), and methylated catechins. However, most monomeric catechins were non-detectable in roots except for EC (Figure 3). PA dimers and trimers comprised of different extension units were found in higher amounts in stems relative to other tissues (Figure S2A–E). They were either not detected or present in very low levels in roots. In contrast, all four identified procyanidin tetramers occurred at the highest level in roots (Figure S2F). A similar finding was reported by Wei et al. in cultivar *Shuchazao*, where they observed a higher accumulation of more condensed PAs in fruits, flowers, and roots. In contrast, young buds and leaves contained more monomeric galloylated catechins [7].

### 2.3. Flavonol Glycosides with Different Aglycone Moieties Displayed Spatial Distribution

Based on UPLC-QTOF MS-based metabolite profiling, we found that *Tieguanyin* tea plants accumulated at least 21 flavonol glycosides, most of which were derivatives of kaempferol (eight compounds), quercetin (six compounds), myricetin (three compounds), and isorhamnetin (two compounds) (Table 1). Some structures were unequivocally identified by comparing with authentic standards, while others were assigned according to MS/MS fragmentation patterns, the neutral loss patterns of specific sugars, UV absorbance, and chromatographic behaviors [42,43], as exemplified in Figures S6 and S7. Among them, the sugar moieties of a few (compounds **44**–**51**) were further acylated to coumaric acid.

The distribution of flavonol glycosides showed intriguing patterns depending on the aglycone moiety (Figure 3 and Figure S8). For example, most kaempferol glycosides were abundant in flowers and young leaves, but scarce in stems and roots (Figure S8A). Quercetin glycosides were detected invariably at the highest level in leaves, and peaked in mature leaves. They were below detection in roots in most cases (Figure S8B). The distribution of myricetin glycosides mirrored that of quercetin glycosides, occurring mainly in the green parts of tea plants and in particular, exhibiting the highest level in mature leaves. They were barely detectable in flowers and roots (Figure S8C). Finally, isorhamnetin glucoside and isorhamnetin coumaroylglucoside were exclusively found in flowers (Figure S8D).

### 2.4. Distribution of Purine Alkaloids and Hydroxycinnamic Acid Amides, Two Classes of Nitrogenous Compounds, Displayed Tissue Specificity

Nitrogen-containing compounds have higher ionization efficiency in ESI^+^. Therefore, two classes of nitrogen-containing metabolites, namely, purine alkaloids and hydroxycinnamic acid amides (HCCAs), were specifically analyzed in this mode. Three major purine alkaloids from the caffeine biosynthetic pathway, including caffeine (compound **61**), theobromine (compound **60**), and 7-methylxanthine (compound **59**), three major purine alkaloids from the caffeine biosynthetic pathway, were detected. The concentrations for all three compounds declined in the same order: buds > young leaves > mature leaves > stems > flowers > roots (Figure 3). A reduction in the caffeine content with the increased leaf age was also noted in other tea cultivars [5,44]. In each tissue, the concentration of caffeine was highest, followed by theobromine and 7-methylxanthine. Roots contained trace amounts of caffeine, theobromine, and almost no 7-methylxanthine.

**Figure 5:**
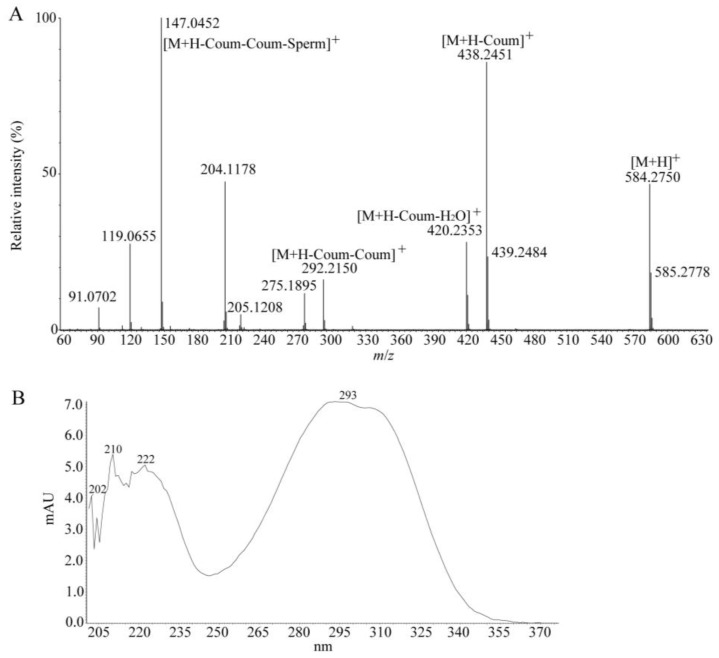
A spermidine derivative (compound **63**) detected in tea flowers. (**A**) CID-MS/MS spectrum of compound **63** in the ESI^+^ mode. (**B**) The UV spectrum of compound **63** extracted from the UPLC-PDA-QTOF MS experiment. mAU, milli absorption unit.

Two HCCAs, including one coumaric-conjugated putrescine (compound **62**) and one coumaric-conjugated spermidine (compound **63**), with the latter being more abundant, were detected almost exclusively in tea floral organs (Table 1). Compound **63** was identified as tricoumaroyl spermidine on the basis of the fragmentation pattern and UV absorbance (Figure 5) in comparison with data available in the literature [40,45]. In MS^2^ analysis, compound **63** with *m*/*z* 584.2750 generated a fragment ion at *m*/*z* 147.0452, corresponding to the coumaric moiety retaining the charge. Major ions at *m*/*z* 438.2451 and 292.2150 could arise from the loss of one coumaric acid and two coumaric acids, respectively, from the molecular ion (Figure 5A). The characteristic UV spectrum showed λ_max_ at 293 nm (Figure 5B), which was in accord with the previous report that the hydroxycinnamoyl-spermidines had a high absorption in the range of 270 nm to 330 nm [46]. Therefore, compound **63** was tentatively assigned as tri-*p*-coumaroylspermidine. Interestingly, this compound was also detected from the tea flowers of cultivar *Yabukita*, and was found to decrease during floral development [40]. Likewise, the fragmentation of compound **62** yielded the diagnostic fragment at *m*/*z* 147.0448, which also corresponded to the coumaric moiety retaining the charge. The fragment ion at *m*/*z* 235.1412 was most likely due to the cleavage of one coumaric acid from the molecular ion, and thus supported the assignment of compound **62** as a putative di-*p*-coumaroylputrescine (Figure S9). As far as we know, this is the first report of the occurrence of this compound in tea flowers.

### 2.5. Differential Amino Acid Profiles among Tea Plant Tissues

To compare amino acid abundance across tea tissues, hydrophilic interaction liquid chromatography (HILIC) tandem mass spectrometry was applied, with the absolute quantification results shown in Table 2. The total amino acid concentration, calculated from the sum of individual amino acid concentrations, was as follows: stems > flowers > mature leaves > young leaves > buds > roots. Theanine, aspartate, glutamate, glutamine, serine, and arginine altogether accounted for 95.9%, 98.6%, 99.2%, and 98.9% of the total amino acids in buds, young leaves, mature leaves, and stems, respectively, and thus were the major amino acids in the green parts of the tea plant.

Theanine, as the most abundant non-protein amino acid in tea, was detected in all of the tissues, but its concentration varied widely, ranging between 19.1–72.9% of the total amino acids. The highest theanine concentration was found in stems, reaching 33.36 mg/g dry weight, followed by mature leaves (10.58 mg/g dry weight). Concentration of theanine in young leaves (10.41 mg/g dry weight) was slightly lower than mature leaves, but higher (*p* < 0.05) than buds (8.36 mg/g dry weight) and flowers (6.32 mg/g dry weight). Roots contained the lowest level of theanine (1.41 mg/g dry weight).

The amino acid profile in the floral organ was distinct from the green parts of tea plants. Notably, concentrations of serine, arginine, asparagine, threonine, histidine, tryptophan, valine, lysine, proline, leucine, phenylalanine, methionine, tyrosine, *γ*-aminobutyric acid, and alanine were significantly higher (*p* < 0.05) in flowers than other parts, suggesting an overall up-regulation of amino acid biosynthesis in flowers (Table 2). Polyamines and CoA-activated hydroxycinnamic acids are two substrates for synthesizing HCCAs [47], in which arginine provides the substrate for the former, while phenylalanine is involved in the production of the latter [47]. Whether the highest occurrence of arginine and phenylalanine is related to the unique occurrence of coumaric-conjugated HCCAs in tea flowers is currently unknown.

## 3. Discussion

The plant kingdom is predicted to produce at least 1,000,000 metabolites [48]. The production, translocation, and hydrolysis of these diverse metabolites are regulated by both intrinsic genetic programs and environmental factors. The limitations imposed by the sensitivity and resolution of analytical techniques, along with rapid metabolite turnovers, have challenged the detection of the majority of metabolites and the systematic studies of their biochemical and biological functions in any single plant species, even in model plants such as Arabidopsis and rice [49,50]. As an economically important beverage crop, tea plants produce arsenals of structurally and biologically diverse nutraceuticals to high levels, among which flavonoids, caffeine, and theanine are best known [51]. Previous studies, which typically targeted one or several classes of target metabolites, reveal the tissue-specific regulation of specialized metabolism in tea plants, as found in other plant species [3,5,6,11,15]. However, the application of untargeted LC-MS-based metabolomics to examine the overall difference in metabolic profiles among tea plant tissues has not been thoroughly performed. With the goal of understanding developmental changes in specialized metabolism, we performed a thorough comparative analysis of six different tissue types in Tieguanyin tea cultivar, which highlighted differences in tissue-specific metabolic features in tea plants. In the present study, thousands of molecular features were simultaneously detected, from which a total of 68 major specialized metabolites belonging to 11 metabolite classes were found to differentially accumulate in different tissues. The comparative results reveal the remarkable diversity of specialized metabolites in tea plants, and provide valuable information to further understand the developmental regulation of their biosynthesis.

### 3.1. The Abundance of Flavonol Glycosides Demonstrates Tissue-specific Variations in Different Plants

Flavonols are among the most abundant flavonoids in plants. Decorative enzymes catalyzing glycosylation, acylation, hydroxylation, and methylation provide important modifications to flavonols, conferring increased structural complexity, enhanced biological activity, as well as improved molecule solubility and stability in *Arabidopsis* and several crop species [52].

In the current study, 21 flavonol glycosides were found to be differentially distributed in different tissues, providing the first insight into the developmental regulation of flavonol glycosides in tea plants. Although the current study is the first report of the tissue-specific distribution of flavonol glycosides in tea plants, it appears as a common trait shared by many plants. Flavonol glycosides have been most thoroughly profiled in *Arabidopsis* tissues, which were discovered to be tightly regulated developmentally in different tissues [42,45]. By UPLC-QTOF MS-based profiling, Yonekura-Sakakibara et al. showed that kaempferol glycosides accounted for 97% of the total flavonoids in leaves, while quercetin glycosides took up 25% of the total flavonoids in floral buds and flowers [53]. In the same study, a higher accumulation of *C*-7 rhamnosylated flavonols in floral buds, in comparison to leaves, roots, and siliques, was found to be well coordinated with the higher expression of a flavonol 7-*O*-rhamnosyltransferase [53]. A more comprehensive flavonol profiling by the same research group revealed that kaempferol 3-*O*-rhamnoside-7-*O*-rhamnoside was one of the major flavonols in leaves, stems, and flowers. In contrast, roots contained very little of this compound, but possessed a high level of quercetin 3-*O*-glucoside-7-*O*-rhamnoside [54]. Moreover, in seeds, quercetin-3-*O*-rhamnoside and a dimer of quercetin-rhamnoside accumulated in the seed coat, while diglycosylated flavonols were only found in the embryo [55]. Significant differences in flavonol compositions were also reported in strawberry and *Compositae* plants [16,56]. For example, quercetin neohesperidoside, kaempferide neohesperidoside, and kaempferol acetylglucoside were detected in the leaf, but not in the flower of *Chrysanthemum morifolium* [56]. In strawberry flowers, dihexose derivatives of kaempferol and quercetin were present mainly in the stamen, but the malonylhexose derivatives of both flavonols were mainly detected in the pistil [16]. The differential production of flavonol glycosides were presumably caused by tissue-specific expression of genes encoding for the synthesis of different flavonols as well as decorative enzymes [57]. Detailed analysis of flavonol levels and gene coexpression is necessary to gain more knowledge on the timing expression of flavonoid biosynthetic genes in *Tieguanyin* tea plant.

Diverse biological roles of flavonols, such as maintaining plant fertility, protecting against UV stress, serving as signaling molecules, functioning as co-pigments, and modulating auxin transport, were documented in a wide range of plant species [58]. Despite their importance, the structure–function relationship for many of these molecules remains largely unknown. In tea plants, different flavonols, along with their different conjugates, presumably play specific roles in the developmental and physiological functions of different tissues, although their exact biological functions are yet to be elucidated. Moreover, flavonols and their glycosyl derivatives could confer astringency to tea infusions at much lower thresholds than catechins, making them important contributors to the flavor property of tea [59]. This, along with flavonol glycosides changing only slightly during tea processing [51], renders the understanding of the tissue-specific distribution of flavonol glycosides among unprocessed tea plant tissues important.

### 3.2. Coumaroyl-Conjugated Hydroxycinnamic Acid Amides (HCCAs) are Unique Flower Metabolites

Widely distributed in the plant kingdom, HCCAs are reported to have important functions in plant adaptation to biotic and abiotic stresses [60]. They are also implicated in some plant growth and developmental processes, including flower formation, sexual differentiation, tuberization, and so on, although the causal relationship is still not conclusive [61]. A wide variety of acylated polyamines have been isolated and identified in the floral parts of different plants. Neutral HCCAs such as di-*p*-coumaroylputrescine, di-*p*-coumaroylspermidine, and tri-*p*-coumaroylspermidine, have been reported in plant reproductive organs, i.e., anthers of fertile maize, male flowers of some *Araceae* species, bee pollen samples, the stamen and pistil of strawberry flowers, and the inflorescence tissues of *Arabidopsis* [16,45,61,62]. Interestingly, in an earlier study, four spermidine derivatives, namely, tricoumaroyl spermidine, feruoyl dicoumaroyl spermidine, coumaroyl diferuoyl spermidine, and triferuoyl spermidine, were found as tea flower constituents in cultivar *Yabukita*. Although we did not dissect the flowers in the current study, spermidine derivatives were previously found to mainly accumulate in the anthers of tea flowers, and as such were presumed to participate in pollen formation [40].

Enzymes that have been identified to date as responsible for synthesizing HCCAs are all acyltransferases belonging to the BAHD family, which utilizes CoA-activated hydroxycinnamic acids and polyamines as substrates [47]. Interestingly, through transcriptome analysis, we identified two unigenes for BAHD acyltransferase, which showed the highest expressions in flowers, but only basal expressions in other tea plant tissues. Functional experimentation is needed to dissect their roles in the HCCA formation in tea flowers.

### 3.3. Occurrence of A-Type PAs is Rare in Tea Plants but Warrants Further Analysis

PAs can be widely found in different parts of various plants, protecting plants against pathogens and herbivores [63]. Similar to monomeric flavan-3-ols, PAs also exhibit a wide array of bioactivities, including antimicrobial, antioxidative, anti-inflammatory, and antihypertensive effects, to name just a few [64]. The most commonly occurring monomeric units of PAs in tea plants are procyanidins, prodelphinidins, and their mixtures, although propelargonidins and galloylated forms of the aforementioned monomeric units also occur [29].

A total of 19 oligomeric PAs, comprising 17 B-type PAs and two A-type PAs, were identified in *Tieguanyin* tea plants. The latter included (E)C-(4→8)-(E)C-(2→7, 4→8)-(E)C and EC-(2*β*→O7, 4*β*→8)-[EC-(4*β*→6)]-EC-(4*β*→8)-EC, the structures of which were tentatively assigned based on fragmentation patterns. Both compounds were found to predominate in roots. Information on A-type PAs detected from tea plants is quite limited. Reported examples only included a dimeric A-type PA isolated from a commercial oolong tea, and a tetrameric A-type PA isolated from fresh tea leaves [65,66]. According to Kumar et al., the occurrence of A-type PAs in tea plants is rare, but is of considerable interest, because they have been implicated to contribute to the beneficial effects of cranberry juice for preventing urinary tract infections [66]. Further chemical analysis of the identified two A-type PAs is required to confirm their structures.

## 4. Materials and Methods

### 4.1. Plant Materials and Sampling

Cuttings of five-year-old cloned tea plants of *C. sinensis* cv. *Tieguanyin* were planted at the tea farm at Anxi Tea Research Institute, Anxi, Fujian Province, China (118°13′ E, 25°08′ N) under the natural environment, where the annual average temperature was 18 °C, and the annual average rainfall was between 1700–1800 mm. The *Tieguanyin* tea plant is an evergreen and perennial shrub with small leaf size, which starts to flower in late October and reaches the full-bloom stage in mid-November. In October 2015, buds, young leaves, mature leaves, new stems (no lignification), flowers, and lateral roots (Figure 1) were harvested with sterile gloves at approximately 10 o′clock in the morning from nine of such tea plants grown under the same cultivation practice. Samples were randomly divided into three groups, with each group containing plant materials collected from three tea plants. Tea plant tissues were washed with tap water to remove attached clay, immediately frozen in liquid nitrogen, brought back to the lab, and stored at −80 °C until analysis. Tissue samples were subjected to UPLC-QTOF MS and UPLC-QqQ MS analyses.

### 4.2. Extraction and UPLC-QTOF MS Analysis

Metabolite extraction was performed according to our previously published protocol [67]. Three biological sample replicates were prepared for each tissue type. One microliter of the metabolite extract was injected into an Acquity UPLC system coupled in tandem to a photodiode array (PDA) detector and a SYNAPT G2-Si HDMS QTOF mass spectrometer (Waters, Milford, MA, USA). Separation was achieved on a Waters Acquity UPLC HSS T3 column (2.1 × 100 mm, 1.8 µm) thermostatted at 40 °C using a gradient from solvent A (water with 0.1% formic acid) to solvent B (acetonitrile with 0.1% formic acid), as previously described [67]. The flow rate was set at 0.3 mL/min. Data were collected in the electrospray ionization (ESI) mode (both ESI^+^ and ESI^−^), scanning from 50–1200 Da. The instrument setup was the same as previously described [67]. Quality control (QC) samples were prepared by mixing an equal amount of each sample to become a combined sample, and were injected every five samples throughout the runs to monitor the instrument performance. The MassLynx software (version 4.1, Waters, Milford, MA, USA) was used to control all of the instruments. Each triplicate tea sample was analyzed once.

### 4.3. Amino Acid Quantitation by UPLC-QqQ MS

To quantify amino acid contents, two microliters of the metabolite extract, with appropriate dilutions within the range of the calibration curve, were injected into an Acquity UPLC system coupled in tandem to a PDA detector and a XEVO TQ-S MS triple quadrupole mass spectrometer (Waters, Milford, MA, USA). Separation was achieved on a Merck SeQuant ZIC-HILIC column (2.1 × 100 mm, 5 µm) thermostatted at 40 °C using a gradient from solvent A (5 mM ammonium acetate) to solvent B (acetonitrile with 0.1% formic acid), as previously described [67]. The flow rate was set at 0.4 mL/min. The instrument setup was same as previously described [67]. Calibration curves generated by injecting increasing concentrations of authentic standards were used to measure the absolute concentrations of amino acids. The MassLynx software (version 4.1, Waters, Milford, MA, USA) was used for instrument control and data acquisition. Each triplicate tea sample was analyzed once.

### 4.4. Data Processing, Metabolite Identification, and Statistical Analysis

Resulting chromatograms from UPLC-QTOF MS were processed using Progenesis QI software (version 2.1, Nonlinear Dynamics, Newcastle upon Tyne, UK) with default settings for peak alignment, normalization, signal integration, and initial compound assignments. Only chromatograms with an elution time between 1–14 min were included in the analysis. Thus, annotation was obtained was used for manual peak identification. Metabolites were identified by comparing accurate masses, MS/MS fragmentation patterns and isotope patterns with authentic standards, online metabolite databases of Metlin [27], MassBank [39], ReSpect [34], KNApSAcK [48] and literature references [26,28,29,30,43]. Each mass spectrum was manually inspected to verify if software-predicted fragments were derived from a single metabolite. UV spectra were used for identification whenever possible.

Samples were acquired in both ESI^+^ and ESI^−^ modes, and therefore, data for each ionization mode were processed in Progenesis QI separately. The software detected 2798 molecular features in ESI^−^ and 3811 molecular features in ESI^+^, which were filtered to include only 732 and 821 single molecular features in respective modes. For comparing the abundances of molecular features, the data matrix consisting of mass features and peak area values was exported from Progenesis QI to Excel. The mean peak area abundance values from three biological replicates of the same tissue type were calculated. Similarities and differences in metabolite signal abundances were compared across tissues. Single molecular features were used as inputs for principal component analysis (PCA) to observe intrinsic metabolite variance between tissues using Progenesis QI extension EZinfo after Pareto scaling. Supervised partial least squared discriminant analysis (PLS-DA) was performed to identify the metabolites that are important for group separation. The data matrix used for PCA and PLS-DA analyses was listed in Supplemental Table S1 (for ESI^−^) and S2 (for ESI^+^). One-way analysis of variance (ANOVA) was carried out using SPSS (version 13.0, Chicago, IL, USA), and differences between means were determined by Tukey′s HSD test. Variable importance in projection (VIP) analysis was performed to evaluate the importance of metabolites. Significantly different metabolites between tissues were selected with VIP > 1 and a *p* value < 0.05. A heat map with hierarchical clustering (Pearson′s correlation, average linkage), after being log2 transformed and normalized to the median level of individual compounds, was generated using MultiExperiment Viewer software (version 4.9.0), which combined data from UPLC-QTOF MS and UPLC-QqQ MS.

### 4.5. Chemicals and Reagents

Acetonitrile (MS grade), methanol (HPLC grade), and formic acid (≥ 98%) were obtained from Sigma-Aldrich (St. Louis, MO, USA). Deionized water was produced by a Milli-Q water purification system (Millipore, Billerica, MA, USA). Standards of EGCG, EGC, C, ECG, EC, GC, rutin, and l-theanine (all with purity ≥ 95%) were obtained from Sigma-Aldrich (St. Louis, MO, USA). EGCG3′′Me (≥95%) and kaempferol glucoside (≥98%) were purchased from ChemFaces (Wuhan, China). Caffeine (≥98%) was obtained from Yuanye Biotechnology Inc. (Shanghai, China). Theobromine (≥99%) and kaempferol glucoside (≥98%) were obtained from BioBioPha Co., Ltd. (Kunming, China). Theogallin (≥95%) was kindly provided by Dr. Qingxi Chen of Fujian Agriculture and Forestry University, China.

## 5. Conclusions

In summary, an UPLC-QTOF MS-based non-targeted metabolomics strategy was applied for the first time to comprehensively compare the specialized metabolite profiles between tea plant tissues. Many metabolite classes, including catechins, PAs, flavonol glycosides, purine alkaloids, HCCAs, and amino acids were found to demonstrate sharp variations among tissue types. The upper part of tea plants abounded in monomeric catechins, whereas the lower part was more enriched in the highly polymerized forms of catechins. The abundance of flavonol glycosides demonstrated tissue specificity depending on the aglycone moiety. Metabolite contents of purine alkaloids and amino acids significantly differed among tissues. Furthermore, two neutral HCCAs, namely, tricoumaroyl spermidine and dicoumaroyl putrescine, were discovered as unique flower metabolites. All of these results suggest that the spatial changes in metabolite levels in tea plants are likely to be developmentally regulated. It also provides a good reference point for formulating a working hypothesis for the future characterization of metabolic functions in tea plants. An interesting aspect for future research would be to further explore gene-metabolite relationships to pinpoint important genes/enzymes and decipher regulatory elements responsible for tissue-specific accumulations of certain metabolites (e.g., flavonol glycosides and HCCAs).

## Figures and Tables

**Table 1:** Metabolites putatively identified in six tissues of *Tieguanyin* tea plants by UPLC-QTOF MS.

Compound	Tentative Assignments	Rt (min)	Detected [M − H]^−^ (*m*/*z*)	Theoretical [M − H]^−^ (*m*/*z*)	Mass Error (ppm)	Formula	MS/MS Fragments	Ref.
**Flavan-3-ols**								
**1**	GC	3.84	305.0670	305.0661	2.95	C_15_H_14_O_7_	219.0664, 179.0353, 167.0351, 139.0399, 125.0245	Authentic standard ^b^
**2**	EGC	4.93	305.0677	305.0661	5.24	C_15_H_14_O_7_	219.0667, 179.0349, 167.0351, 139.0402, 125.0245	Authentic standard ^b^
**3**	C	5.36	289.0719	289.0712	2.42	C_15_H_14_O_6_	245.0822, 203.0714, 125.0246	Authentic standard ^b^
**4**	EC	6.27	289.0721	289.0712	3.11	C_15_H_14_O_6_	245.0819, 203.0714, 123.0451	Authentic standard ^b^
**5**	EGCG	6.35	457.0797	457.0771	5.69	C_22_H_18_O_11_	305.0669, 169.0160, 125.0247	Authentic standard ^b^
**6**	EGCG3”Me	7.42	471.0934	471.0927	1.49	C_23_H_20_O_11_	305.0674, 287.0568, 183.0304, 161.0251, 125.0247	Authentic standard ^b^
**7**	ECG	7.86	441.0828	441.0822	1.36	C_22_H_18_O_10_	331.0462, 289.0720, 245.0819, 169.0147, 125.0245	Authentic standard ^b^
**8**	ECG3”Me	8.92	455.0960	455.0978	−3.96	C_23_H_20_O_10_	289.0721, 183.0302	[26]
**9**	epiafzelechin 3-gallate	8.97	425.0881	425.0873	1.88	C_22_H_18_O_9_	273.0761, 169.0140, 151.0029, 137.0245, 125.0243	[27]
**Proanthocyanidins**								
**10**	prodelphinidin B isomer 1	3.34	609.1246	609.1244	0.33	C_30_H_26_O_14_	483.0947, 441.0827, 423.0717, 305.0667	[26]
**11**	prodelphinidin B isomer 2	4.11	609.1249	609.1244	0.82	C_30_H_26_O_14_	483.0932, 441.0822, 423.0716, 305.0668	[26]
**12**	EC-GC dimer	4.80	593.1300	593.1295	0.84	C_30_H_26_O_13_	425.0857, 423.0707, 305.0670, 289.0717, 125.0245	[27]
**13**	prodelphinidin B-2 (or 4) 3′-*O*-gallate	5.11	761.1352	761.1354	−0.26	C_37_H_30_O_18_	609.1236, 591.1135, 577.1348, 423.0718	[26]
**14**	procyanidin trimer (B type) isomer 1	5.25	865.1962	865.1980	−2.08	C_45_H_38_O_18_	695.1369, 577.1319, 451.1034, 287.0553	[16]
**15**	procyanidin trimer (B type) isomer 2	5.52	865.1966	865.1980	−1.62	C_45_H_38_O_18_	695.1389, 575.1181, 451.0982, 287.0561	[16]
**16**	procyanidin dimer (B type) isomer 1	5.68	577.1349	577.1346	0.52	C_30_H_26_O_12_	451.1031, 425.0873, 407.0766, 289.0717, 125.0243	[28]
**17**	procyanidin B2	5.78	577.1326	577.1346	−3.47	C_30_H_26_O_12_	451.1022, 425.0864, 407.0763, 289.0713, 125.0243	Authentic standard ^b^
**18**	procyanidin tetramer (B type) isomer 1	5.88	1153.2599	1153.2614	−1.30	C_60_H_50_O_24_	1027.2271, 865.1966, 576.1259, 575.1178, 287.0546	[29]
**19**	procyanidin tetramer (B type) isomer 2	5.97	1153.2599	1153.2614	−1.30	C_60_H_50_O_24_	1027.2234, 577.1329, 575.1175, 287.0557	[29]
**20**	procyanidin trimer (B type) isomer 3	5.99	865.1957	865.1980	−2.66	C_45_H_38_O_18_	739.1646, 713.1482, 695.1387, 577.1292, 451.1020, 423.0711, 407.0760, 287.0557	[16]
**21**	EGC-ECG dimer	6.04	745.1394	745.1405	−1.48	C_37_H_30_O_17_	593.1265, 423.0709, 407.0763, 169.0137	[30]
**22**	(E)C-(4→8)-(E)C-(2→7, 4→8)-(E)C	6.49	863.1814	863.1823	−1.04	C_45_H_36_O_18_	711.1324, 693.1232, 575.1180, 573.1035, 287.0559, 285.0392	[31]
**23**	procyanidin tetramer isomer 3 (B type)	6.72	1153.2589	1153.2614	−2.17	C_60_H_50_O_24_	865.1940,575.1193,287.0553	[29]
**24**	3-galloylprocyanidin B1/3′-galloylprocyanidin B2 isomer 1	6.78	729.1455	729.1456	−0.14	C_37_H_30_O_16_	603.1136, 441.0826, 407.0768, 289.0716, 125.0244	[30]
**25**	parameritannin A-1	6.92	1153.2572 ^a^	1153.2614 ^a^	−3.64	C_60_H_48_O_24_	1001.2155, 866.2023, 579.1450, 577.1265	[32,33]
**26**	epiafzelechin 3-*O*-gallate-(4β→6)-epigallocatechin 3-*O*-gallate	7.50	883.1722 ^a^	883.1722 ^a^	0.00	C_44_H_34_O_20_	409.0919, 271.0606, 153.0190	[27]
**27**	epiafzelechin epicatechin 3,3′-digallate	8.14	867.1774 ^a^	867.1773 ^a^	0.12	C_44_H_34_O_19_	547.1236, 393.0977, 299.0561, 267.0661, 255.0660, 243.0659, 231.0663	[27]
**28**	3-galloylprocyanidin B1/3′-galloylprocyanidin B2 isomer 2	8.86	729.1455	729.1456	−0.14	C_37_H_30_O_16_	603.1140, 577.1115, 441.0829, 417.1560, 407.0777	[30]
**Flavonol/Flavone Glycosides**								
**29**	isovitexin glucoside	6.08	595.1653 ^a^	595.1663 ^a^	−1.68	C_27_H_30_O_15_	473.1142, 433.1129, 313.0711	[34,35]
**30**	apigenin 6-*C*-glucoside 8-*C*-arabinoside	6.91	563.1402	563.1401	0.18	C_26_H_28_O_14_	545.1306, 503.1185, 473.1089, 443.1089, 383.0767, 353.0663	[35]
**31**	myricetin 3-robinobioside (or 3-neohesperidoside)	6.93	627.1556 ^a^	627.1561 ^a^	−0.80	C_27_H_30_O_17_	481.1028, 319.0457	[27]
**32**	myricetin 3-galactoside	7.02	479.0828	479.0826	0.42	C_21_H_20_O_13_	317.0284, 316.0232, 271.0249	[26]
**33**	myricetin 3′-glucoside	7.11	479.0828	479.0826	0.42	C_21_H_20_O_13_	317.0283, 316.0232, 271.0250	[26]
**34**	quercetin 3-*O*-galactosyl rutinoside	7.21	771.1981	771.1984	−0.39	C_33_H_40_O_21_	611.1627, 465.1064, 301.0348, 300.0270	[27]
**35**	camellianin B	7.68	577.1551	577.1557	−1.04	C_27_H_30_O_14_	433.1134, 313.0717, 269.0445	[36]
**36**	rutin	7.70	609.1450	609.1456	−1.85	C_27_H_30_O_16_	301.0343, 300.0280	Authentic standard ^b^
**37**	kaempferol 3-*O*-galactosyl rutinoside	7.72	757.2177 ^a^	757.2191 ^a^	0.00	C_33_H_40_O_20_	595.1652, 449.1080, 287.0557	[26]
**38**	tricetin	7.89	303.0506 ^a^	303.0505 ^a^	0.33	C_15_H_10_O_7_	285.0410	[27]
**39**	kaempferol 3-*O*-glucosyl rutinoside	8.00	757.2187 ^a^	757.2191 ^a^	−0.53	C_33_H_40_O_20_	595.1661, 449.1079, 287.0563	[26]
**40**	kaempferol 3-*O*-rutinoside	8.43	595.1667 ^a^	595.1663 ^a^	0.67	C_27_H_30_O_15_	503.0271, 449.1084, 287.0562	[27]
**41**	kaempferol galactoside	8.51	447.0928	447.0927	0.22	C_21_H_20_O_11_	285.0376, 284.0328	[26]
**42**	isorhamnetin glucoside	8.65	477.1038	477.1033	1.12	C_22_H_22_O_12_	357.1347, 315.0504, 314.0435, 300.0271, 299.0203	[28]
**43**	kaempferol glucoside	8.78	447.0930	447.0927	0.67	C_21_H_20_O_11_	285.0393, 284.0333	Authentic standard ^b^
**44**	capilliposide I isomer 1	9.93	1063.2920	1063.2931	−1.03	C_48_H_56_O_27_	917.2346, 771.1968,753.1868, 615.1923,609.1423, 531.1428, 458.1134, 447.0933, 301.0351, 300.0273	[37]
**45**	capilliposide II isomer 1	10.18	1049.3125 ^a^	1049.3138 ^a^	−1.24	C_48_H_56_O_26_	887.2597, 741.2037, 595.1495, 287.0557	[37]
**46**	quercetin 3-(4”-(*E*)-*p*-coumaroylrobinobioside)-7-rhamnoside isomer 1	10.24	903.2554 ^a^	903.2559 ^a^	−0.55	C_42_H_46_O_22_	757.1984, 449.1078, 303.0508, 147.0448	[27]
**47**	capilliposide I isomer 2	10.59	1065.3074 ^a^	1065.3087 ^a^	−1.22	C_48_H_56_O_27_	919.2526, 617.2090, 449.1088, 303.0505	[37]
**48**	capilliposide II isomer 2	10.87	1049.3136 ^a^	1049.3138 ^a^	−0.19	C_48_H_56_O_26_	887.2601, 741.2042, 595.1545, 287.0559	[37]
**49**	quercetin 3-(4′′-(*E*)-*p*-coumaroyl robinobioside)-7-rhamnoside isomer 2	10.91	903.2546 ^a^	903.2559 ^a^	−1.44	C_42_H_46_O_22_	757.1981, 449.1070, 303.0505, 147.0449	[27]
**50**	isorhamnetin 3 (or 7)-(6′′-*p*-coumaroylglucoside)	10.98	623.1400	623.1401	−0.16	C_31_H_28_O_14_	477.1068, 315.0508, 300.0271, 299.0200	[27]
**51**	2′′-*O*-trans-*p*-coumaroylastragalin	11.68	593.1296	593.1295	0.17	C_30_H_26_O_13_	447.0938, 285.0407, 284.0325	[28]
**Phenolic Acids**								
**52**	theogallin	2.90	343.0679	343.0665	4.08	C_14_H_16_O_10_	191.0564	Authentic standard ^b^
**53**	3-*p*-coumaroylquinic acid	5.18	337.0931	337.0923	2.37	C_16_H_18_O_8_	163.0403	[26]
**54**	5-*p*-coumaroylquinic acid	6.41	337.0930	337.0923	2.08	C_16_H_18_O_8_	173.0459	[26]
**Hydrolysable Tannins**								
**55**	monogalloyl glucose	2.45	331.0672	331.0665	2.11	C_13_H_16_O_10_	271.0461, 211.0248, 169.0144, 151.0040, 125.0244	[16]
**56**	methyl 6-*O*-galloyl-*β*-d-glucose	3.66	345.0827	345.0822	1.45	C_14_H_18_O_10_	225.0406, 183.0299	[27]
**57**	digalloyl glucose isomer 1	4.76	483.0780	483.0775	1.04	C_20_H_20_O_14_	313.0578, 169.0139	[38]
**58**	digalloyl glucose isomer 2	5.01	483.0779	483.0775	0.83	C_20_H_20_O_14_	313.0559, 169.0142	[38]
**Alkaloids**								
**59**	7-methylxanthine	2.84	167.0570 ^a^	167.0569 ^a^	0.60	C_6_H_6_N_4_O_2_	124.0514	[39]
**60**	theobromine	3.80	181.0729 ^a^	181.0726 ^a^	1.66	C_7_H_8_N_4_O_2_	163.0622, 138.0674	Authentic standard ^b^
**61**	caffeine	5.60	195.0885 ^a^	195.0882 ^a^	1.54	C_8_H_10_N_4_O_2_	138.0673	Authentic standard ^b^
**Hydroxycinnamic Acid Amides**								
**62**	di-*p*-coumaroylputrescine	10.33	381.1816 ^a^	381.1814 ^a^	0.53	C_22_H_24_N_2_O_4_	235.1412, 218.1179, 147.0448, 119.0653, 91.0701	[27]
**63**	tri-*p*-coumaroylspermidine	12.08	584.2750 ^a^	584.2761 ^a^	−1.88	C_34_H_3_7__N_3_6__O	438.2451, 420.2353, 292.2150, 275.1895, 205.1208, 204.1178, 147.0609, 119.0655, 91.0702	[40]
**Amino Acids**								
**64**	theanine	1.43	173.0935	173.0926	5.20	C_7_H_14_N_2_O_3_	155.0830, 128.0354	Authentic standard ^b^
**Aromatic Alcohol Glycosides**								
**65**	phenylethyl primeveroside	7.10	415.1599	415.1604	−1.20	C_19_H_28_O_10_	283.1177, 149.0448	[27]
**Terpenoid Glycosides**								
**66**	linalool oxide primeveroside	8.59	463.2166	463.2179	−2.81	C_21_H_36_O_11_	331.1761	[27]
**67**	linalool primeveroside isomer 1	11.25	447.2234	447.2230	0.89	C_21_H_36_O_10_	315.1805	[27]
**68**	linalool primeveroside isomer 2	11.53	447.2233	447.2230	0.67	C_21_H_36_O_10_	421.1703	[27]

^a^ [M + H]^+^. ^b^ This letter indicates that identification of the compound was confirmed by the authentic standard.

**Table 2:** Abundance (mg/g dry weight) of amino acids in tea plant tissues.

Amino Acids	Bud	Young Leaf	Mature Leaf	Stem	Flower	Root
Theanine	8.36 ± 0.41 c	10.41 ± 1.07 b	10.58 ± 0.91 b	33.36 ± 0.25 a	6.32 ± 0.81 d	1.41 ± 0.08 e
Aspartate	8.27 ± 0.37 ab	7.08 ± 0.55 b	7.81 ± 0.25 ab	9.71 ± 1.60 a	7.69 ± 0.74 ab	ND
Glutamate	1.88 ± 0.06 c	2.55 ± 0.14 b	3.01 ± 0.10 a	2.55 ± 0.13 b	1.74 ± 0.07 c	0.26 ± 0.01 d
Glutamine	0.99 ± 0.02 c	0.58 ± 0.04 d	0.35 ± 0.02 de	6.15 ± 0.27 a	2.77 ± 0.14 b	0.03 ± 0.00 e
Serine	0.96 ± 0.03 b	0.75 ± 0.06 bc	0.42 ± 0.03 cd	0.46 ± 0.07 c	2.79 ± 0.37 a	0.03 ± 0.01 d
Arginine	0.47 ± 0.02 c	0.70 ± 0.05 b	0.09 ± 0.01 d	0.45 ± 0.01 c	1.55 ± 0.10 a	0.05 ± 0.00 d
Asparagine	0.41 ± 0.01 b	0.03 ± 0.00 d	0.01 ± 0.00 d	0.10 ± 0.02 c	0.98 ± 0.04 a	0.01 ± 0.01 d
Threonine	0.09 ± 0.00 b	0.03 ± 0.01 cd	0.02 ± 0.00 d	0.08 ± 0.01 bc	0.34 ± 0.05 a	ND
Histidine	0.08 ± 0.00 c	0.03 ± 0.00 d	0.01 ± 0.00 de	0.10 ± 0.01 b	0.34 ± 0.02 a	0.01 ± 0.00 e
Tryptophan	0.08 ± 0.00 b	0.02 ± 0.00 e	ND	0.05 ± 0.00 c	0.26 ± 0.00 a	0.03 ± 0.00 d
Valine	0.07 ± 0.00 b	0.06 ± 0.00 b	0.03 ± 0.00 c	0.06 ± 0.00 b	0.35 ± 0.02 a	0.01 ± 0.00 d
Lysine	0.06 ± 0.00 c	0.06 ± 0.00 c	0.03 ± 0.00 d	0.10 ± 0.01 b	0.28 ± 0.01 a	0.01 ± 0.00 e
Proline	0.05 ± 0.00 b	0.03 ± 0.00 b	0.02 ± 0.00 b	0.02 ± 0.00 b	4.36 ± 0.06 a	ND
Leucine	0.02 ± 0.00 b	0.01 ± 0.00 b	0.02 ± 0.00 b	0.02 ± 0.00 b	0.14 ± 0.01 a	ND
Isoleucine	0.02 ± 0.00 b	0.01 ± 0.00 bc	ND	0.01 ± 0.00 bc	0.36 ± 0.01 a	ND
Phenylalanine	0.01 ± 0.00 c	0.01 ± 0.00 c	0.02 ± 0.00 c	0.07 ± 0.00 b	1.30 ± 0.03 a	ND
Methionine	0.01 ± 0.00 b	ND	ND	0.01 ± 0.00 b	0.08 ± 0.01 a	ND
Tyrosine	ND	ND	ND	0.01 ± 0.00 b	0.13 ± 0.01 a	ND
*γ*-Aminobutyric acid	ND	ND	ND	0.03 ± 0.00 c	0.20 ± 0.01 a	0.06 ± 0.00 b
Alanine	ND	0.01 ± 0.01 b	ND	ND	1.15 ± 0.25 a	ND
total	21.82 ± 0.94 c	22.38 ± 1.94 c	22.43 ± 1.32 c	53.35 ± 2.40 a	33.14 ± 2.76 b	1.92 ± 0.11 d

Results are expressed as mean ± standard deviation (n = 3). Means with different letters in row are significantly different according to Tukey′s HSD (honestly significant difference) test (*p* < 0.05). ND = non-detectable.

## Supplementary Materials

The Supplementary Materials are available online. Figure S1 UPLC-QTOF MS total ion chromatograms in ESI^−^ of six tea tissues. Figure S2 Mean peak area abundance values (±SD) of (A) procyanidin dimers, (B) prodelphinidin dimers, (C) procyanidin-prodelphinidin dimers, (D) propelargonidin dimers, (E) procyanidin trimers and (F) procyanidin tetramers in tea plant tissues. Figure S3 Reconstructed ion chromatograms and MS/MS fragmentation of putative procyanidin trimers. Figure S4 Reconstructed ion chromatograms and MS/MS fragmentation of putative procyanidin tetramers. Figure S5 CID-MS/MS spectrum of compound **22** in the ESI^−^ mode. Figure S6 Reconstructed ion chromatograms and MS/MS fragmentation of kaempferol hexose-deoxyhexose-hexose. Figure S7 Reconstructed ion chromatograms and MS/MS fragmentation of capilliposide I. Figure S8 Mean peak area abundance values (± SD) of (A) kaempferol glycosides, (B) quercetin glycosides, (C) myricetin glycosides and (D) isorhamnetin glycosides in tea plant tissues. Figure S9 CID-MS/MS spectrum of compound **62**, a putative di-*p*-coumaroylputrescine, detected from tea flowers. Table S1 Filtered and normalized PCA data matrix generated from UPLC-QTOF MS in ESI^−^. Table S2 Filtered and normalized PCA data matrix generated from UPLC-QTOF MS in ESI^+^.
