# Supplementary material for: Insights into Tissue-specific Specialized Metabolism in Tieguanyin Tea Cultivar by Untargeted Metabolomics
Source: Molecules. 2018 Jul 21;23(7):1817. doi: 10.3390/molecules23071817 (PMC6099842; doi:10.3390/molecules23071817)
Supplement: Supplementary file 1 [file molecules-23-01817-s001.zip › Supplemental materials_07182018.docx]

Supplemental Materials

Novel Insights into Tissue-specific Specialized Metabolism in *Tieguanyin* Tea Cultivar by Untargeted Metabolomics

Si Chen^1,2#^, Jun Lin^2#^, Huihui Liu^1,2^, Zhihong Gong^2^, Xiaxia Wang^2^, Meihong Li^1,2^, Asaph Aharoni^3^, Zhenbiao Yang^2,4^, Xiaomin Yu^2*^

^1^ College of Horticulture, Fujian Agriculture and Forestry University, Fuzhou 350002, China

^2^ FAFU-UCR Joint Center for Horticultural Biology and Metabolomics, Fujian Provincial Key Laboratory of Haixia Applied Plant Systems Biology, Fujian Agriculture and Forestry University, Fuzhou 350002, China

^3^ Department of Plant & Environmental Sciences, Weizmann Institute of Science, P. O. Box 26, Rehovot 7610001, Israel

^4^ Center for Plant Cell Biology, Institute for Integrative Genome Biology, and Department of Botany and Plant Sciences, University of California, Riverside, CA 92521, USA

***** Correspondence: xmyu0616@fafu.edu.cn; Tel.: +86-591-8639-1591

# S.C. and J.L. contributed equally to this work.


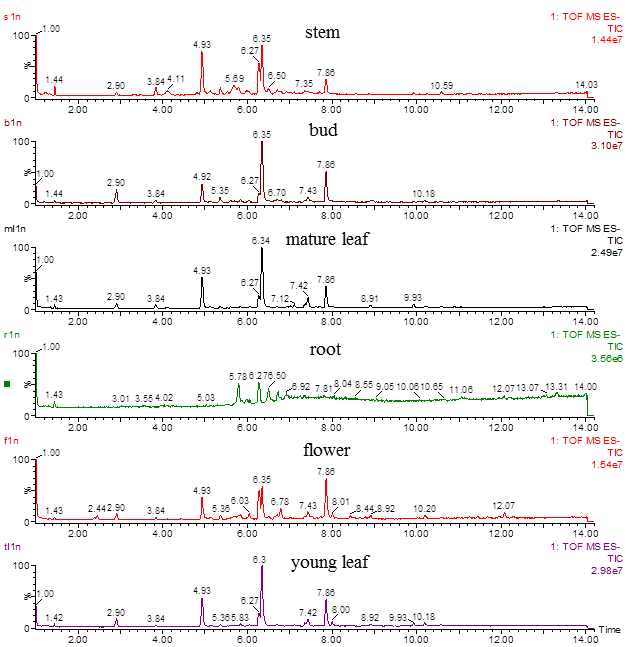


**Figure S1**. UPLC-QTOF MS total ion chromatograms in ESI^-^ of six tea tissues.


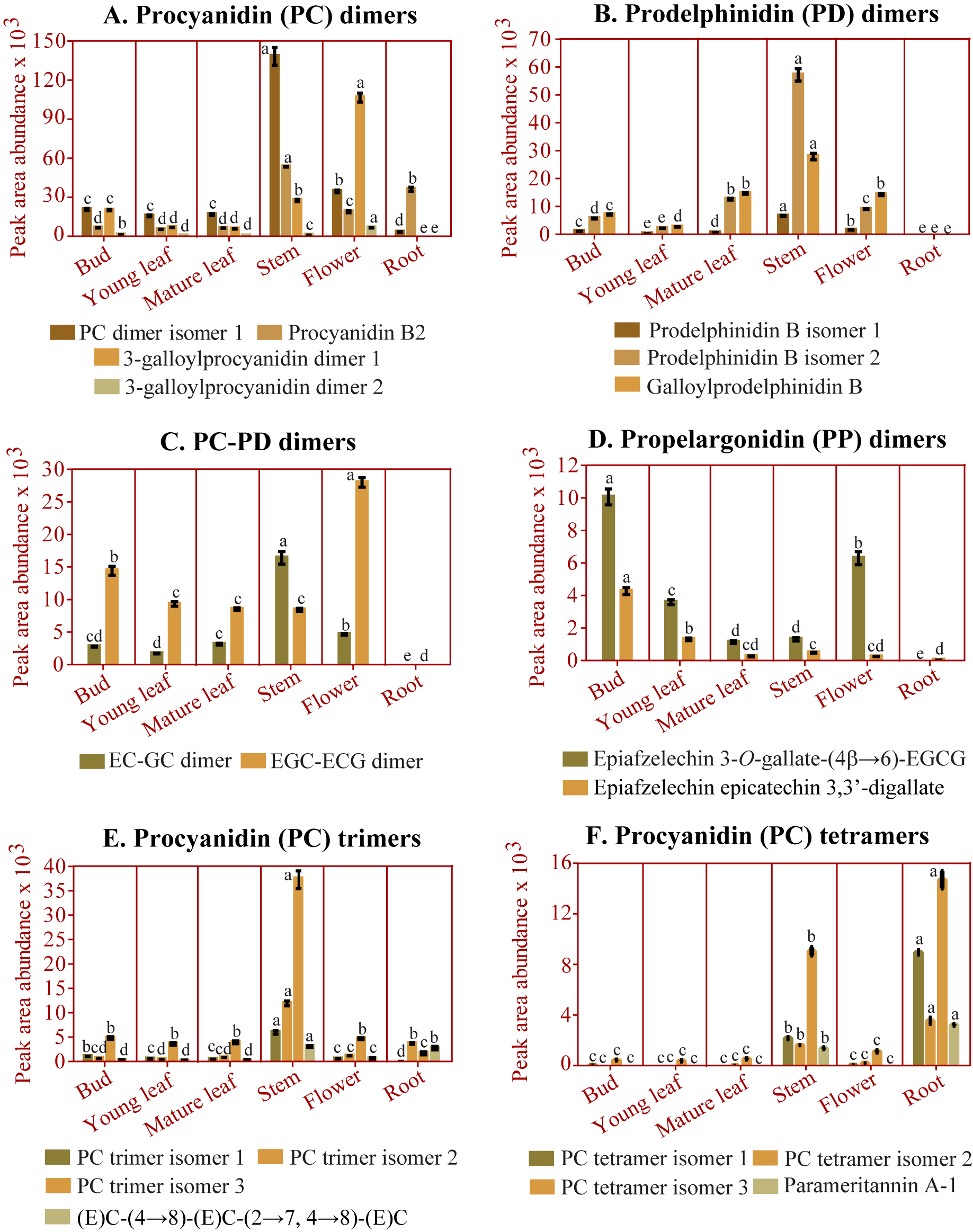


**Figure S2**. Mean peak area abundance values (±SD) of (A) procyanidin dimers, (B) prodelphinidin dimers, (C) procyanidin-prodelphinidin dimers, (D) propelargonidin dimers, (E) procyanidin trimers and (F) procyanidin tetramers in tea plant tissues. Different letters on top of the vertical bars indicate significant differences among the samples, which were determined by Tukey’s HSD test at *p*<0.05.


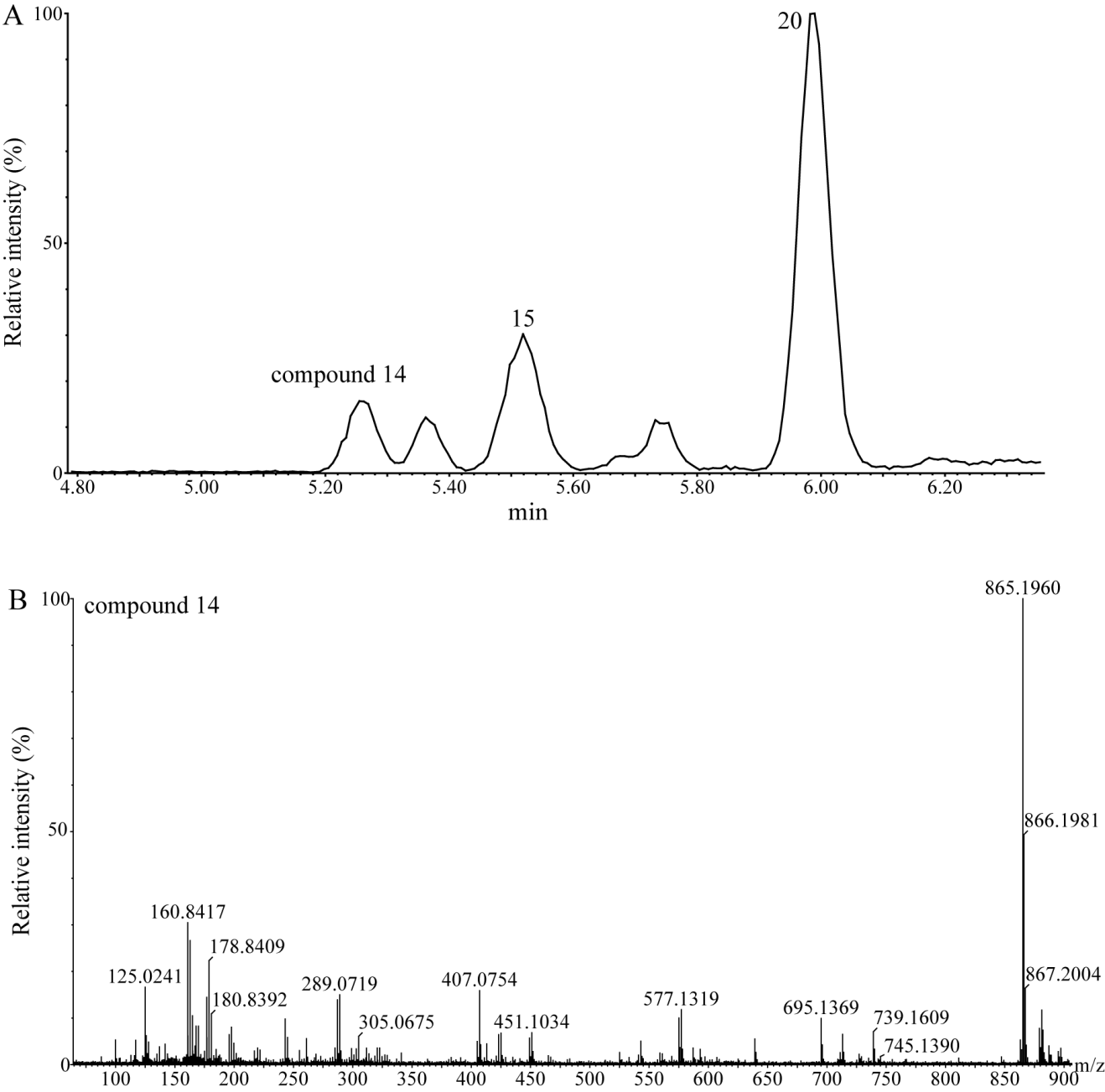


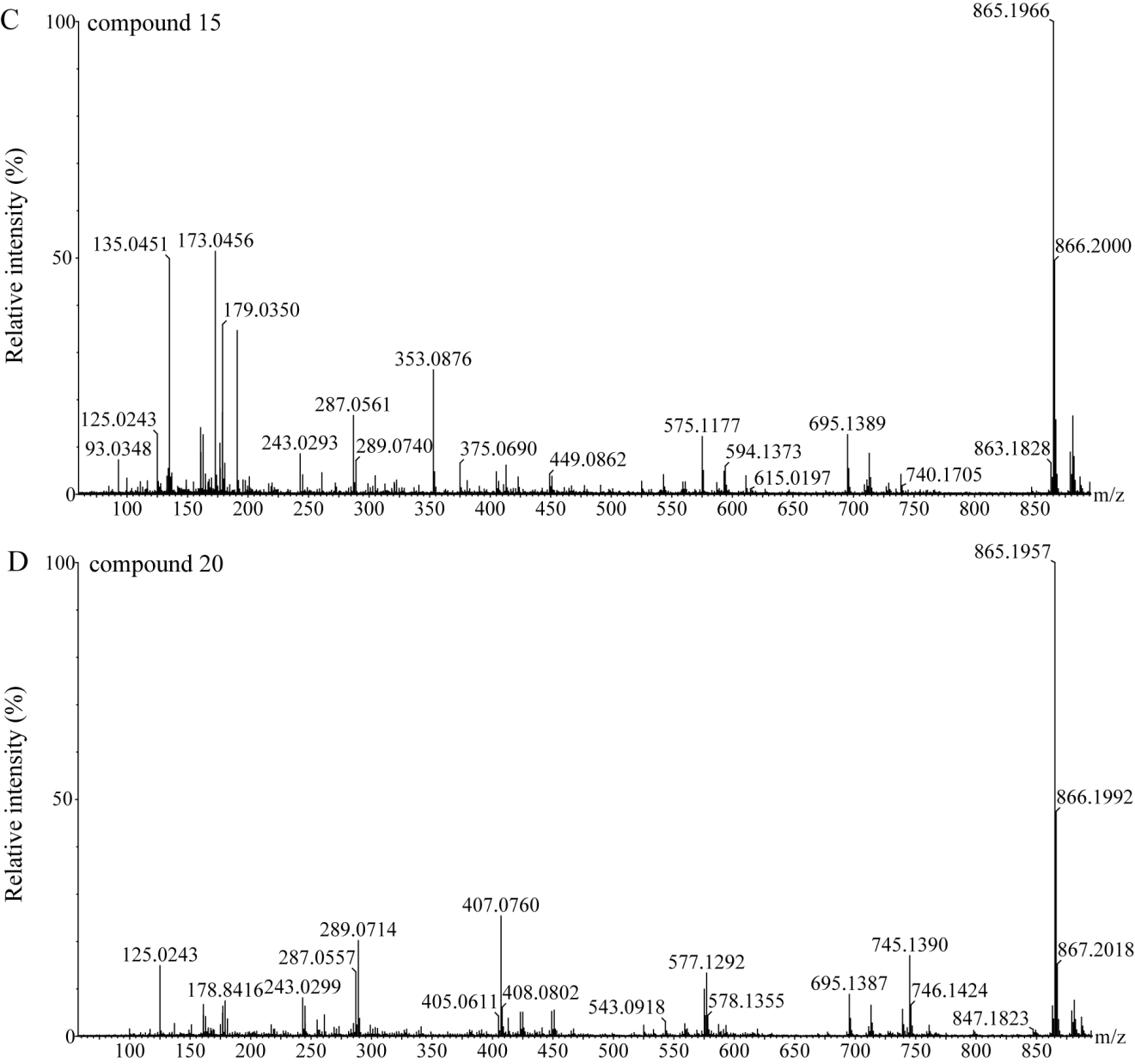


**Figure S3**. Reconstructed ion chromatograms and MS/MS fragmentation of putative procyanidin trimers. (A) Reconstructed ion chromatograms of compounds 14, 15 and 20. (B) MS/MS spectrum of compound 14 in ESI^-^. (C) MS/MS spectrum of compound 15 in ESI^-^. (D) MS/MS spectrum of compound 20 in ESI^-^.


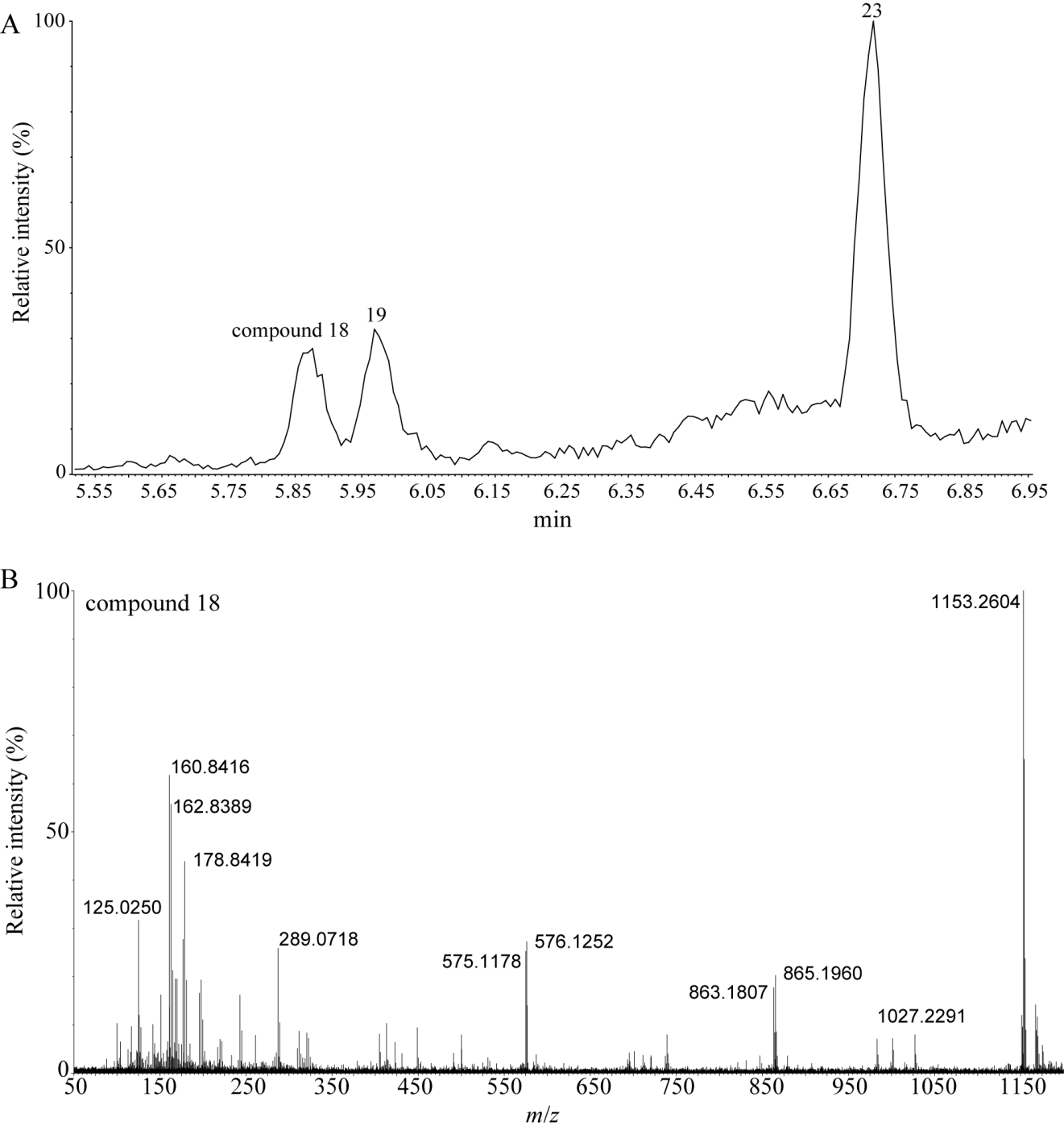


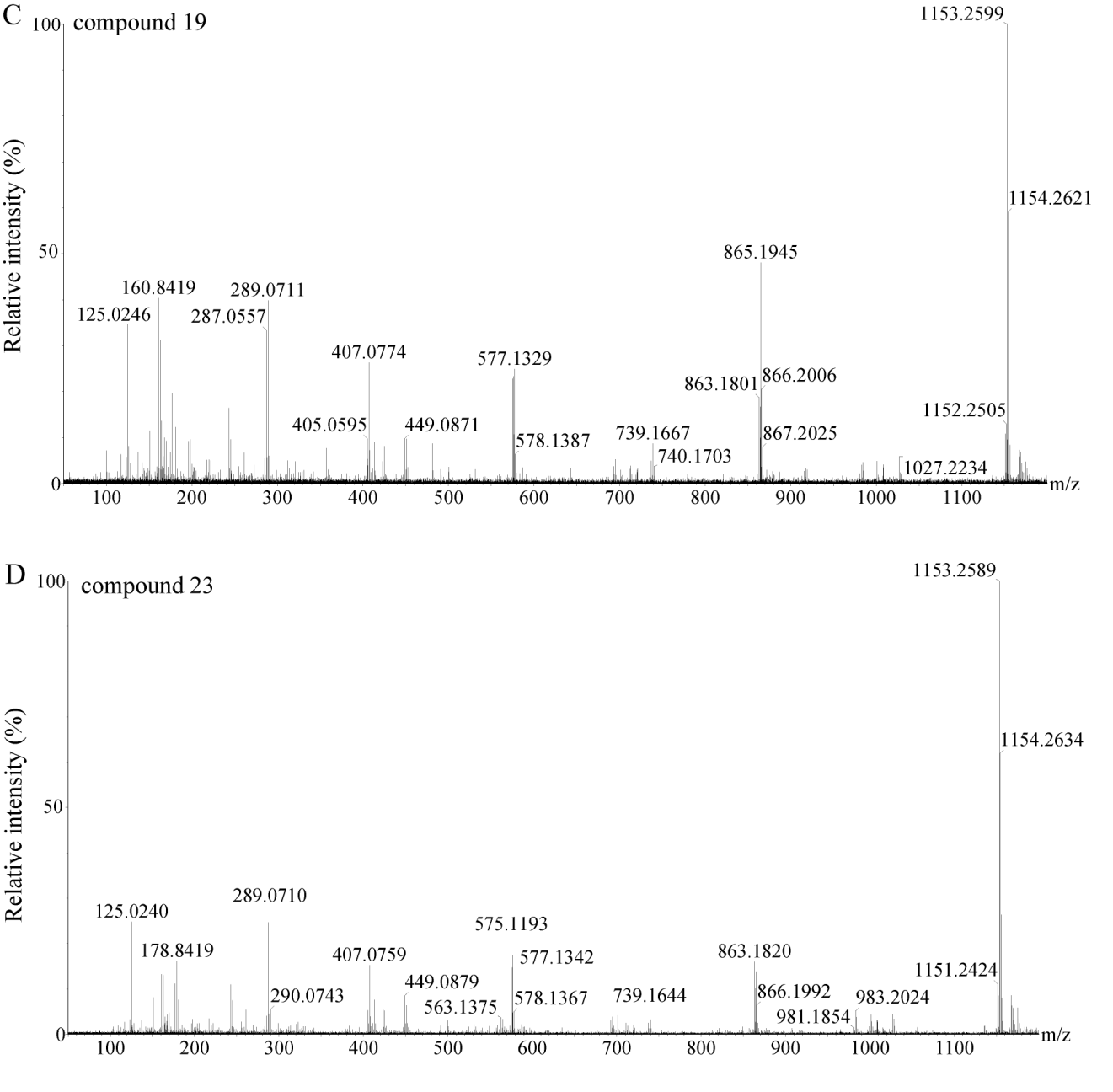


**Figure S4**. Reconstructed ion chromatograms and MS/MS fragmentation of putative procyanidin tetramers. (A) Reconstructed ion chromatograms of compounds 18, 19 and 23. (B) MS/MS spectrum of compound 18 in ESI^-^. (C) MS/MS spectrum of compound 19 in ESI^-^. (D) MS/MS spectrum of compound 23 in ESI^-^.


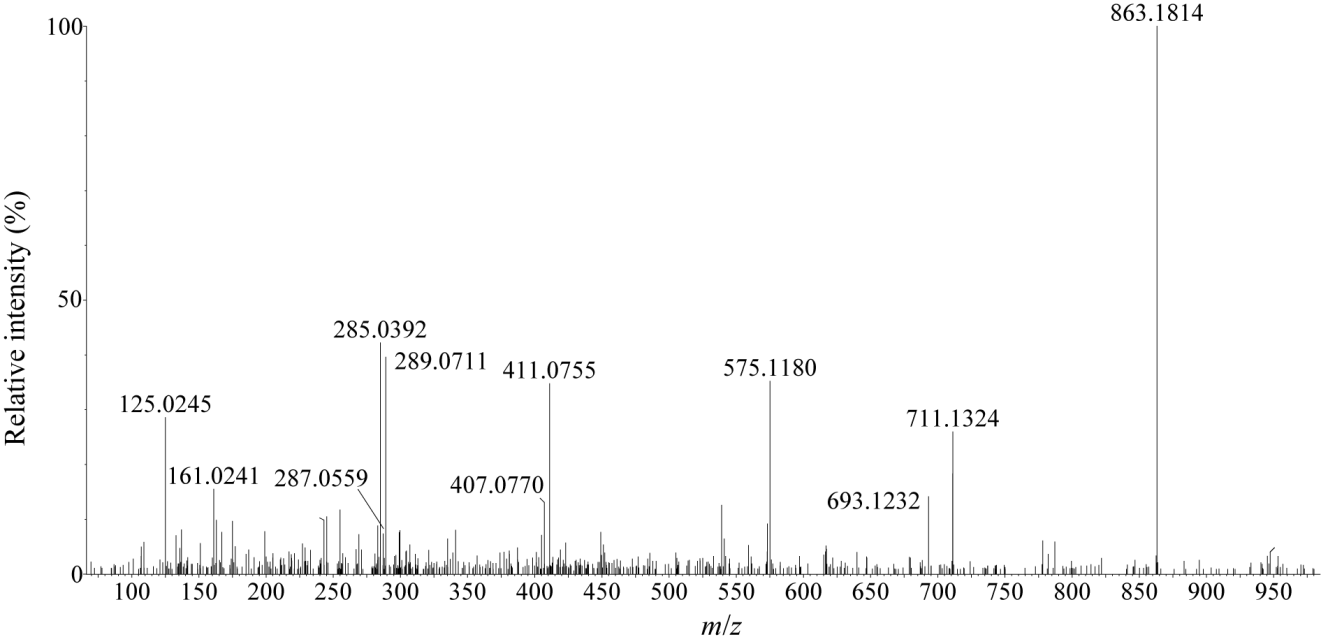


**Figure S5.** CID-MS/MS spectrum of compound 22 in the ESI^-^ mode.


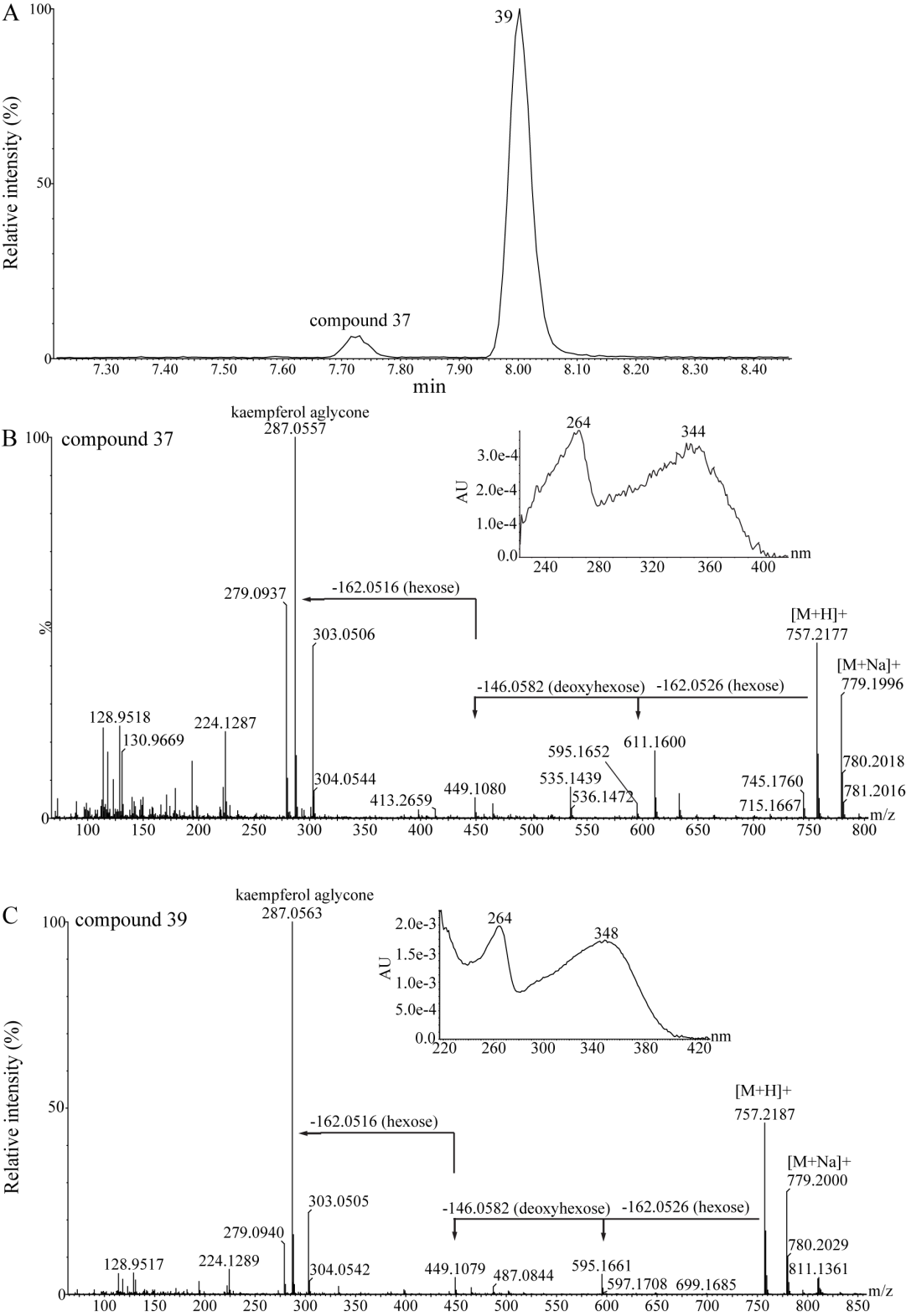


**Figure S6.** Reconstructed ion chromatograms and MS/MS fragmentation of kaempferol hexose-deoxyhexose-hexose. (A) Reconstructed ion chromatograms of compounds 37 and 39. (B) MS/MS spectrum of compound 37 in ESI^+^. (C) MS/MS spectrum of compound 39 in ESI^+^. Inlets showed the UV spectra of compounds 37 (B) and 39 (C). AU, absorption unit.


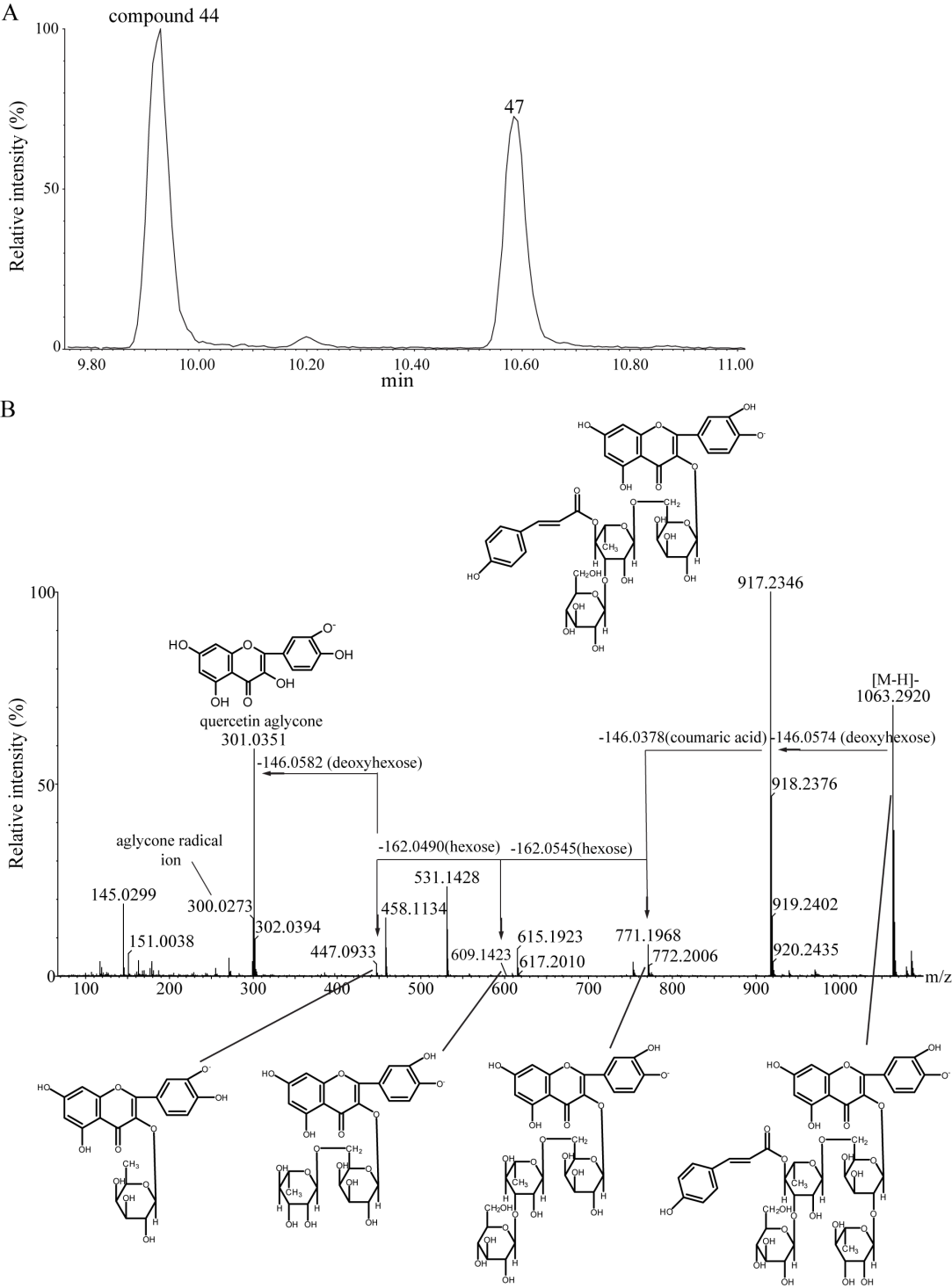


**Figure S7.** Reconstructed ion chromatograms and MS/MS fragmentation of capilliposide I (also known as quercetin 3-*O*-[β-D-glucopyranosyl (1-3)-(4-coumaroyl)-α-L-rhamopyransoyl (1-6)]-[α-L-rhamnopyranosyl (1-2)]-β-D-galactopyranoside). (A) Reconstructed ion chromatograms of compounds 44 and 47. (B) MS/MS spectrum of compound 44 in ESI^-^.


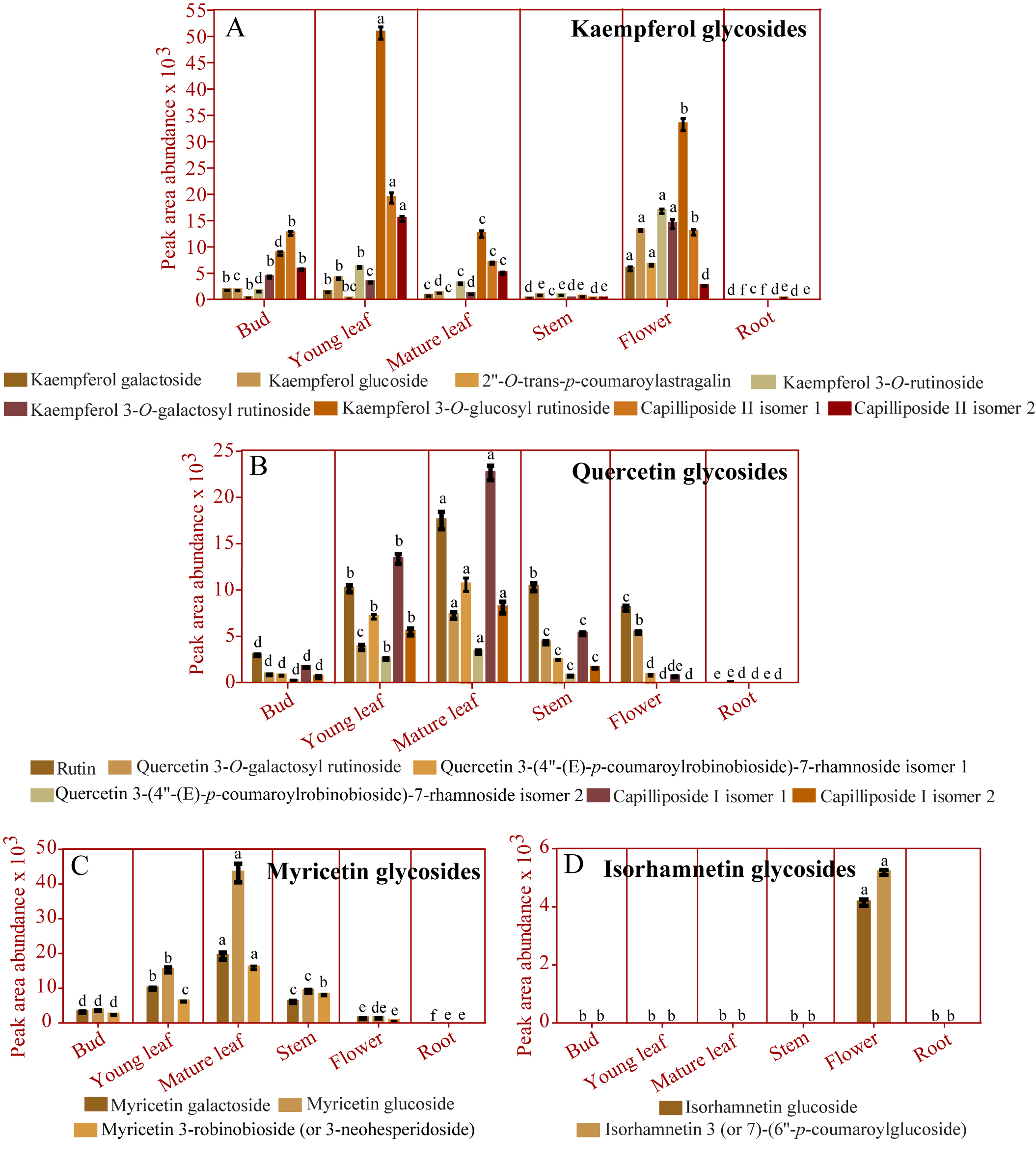


**Figure S8.** Mean peak area abundance values (±SD) of (**A**) kaempferol glycosides, (**B**) quercetin glycosides, (**C**) myricetin glycosides and (**D**) isorhamnetin glycosides in tea plant tissues. Different letters on top of the vertical bars indicate significant differences among the samples, which were determined by Tukey’s HSD test at *p*<0.05.


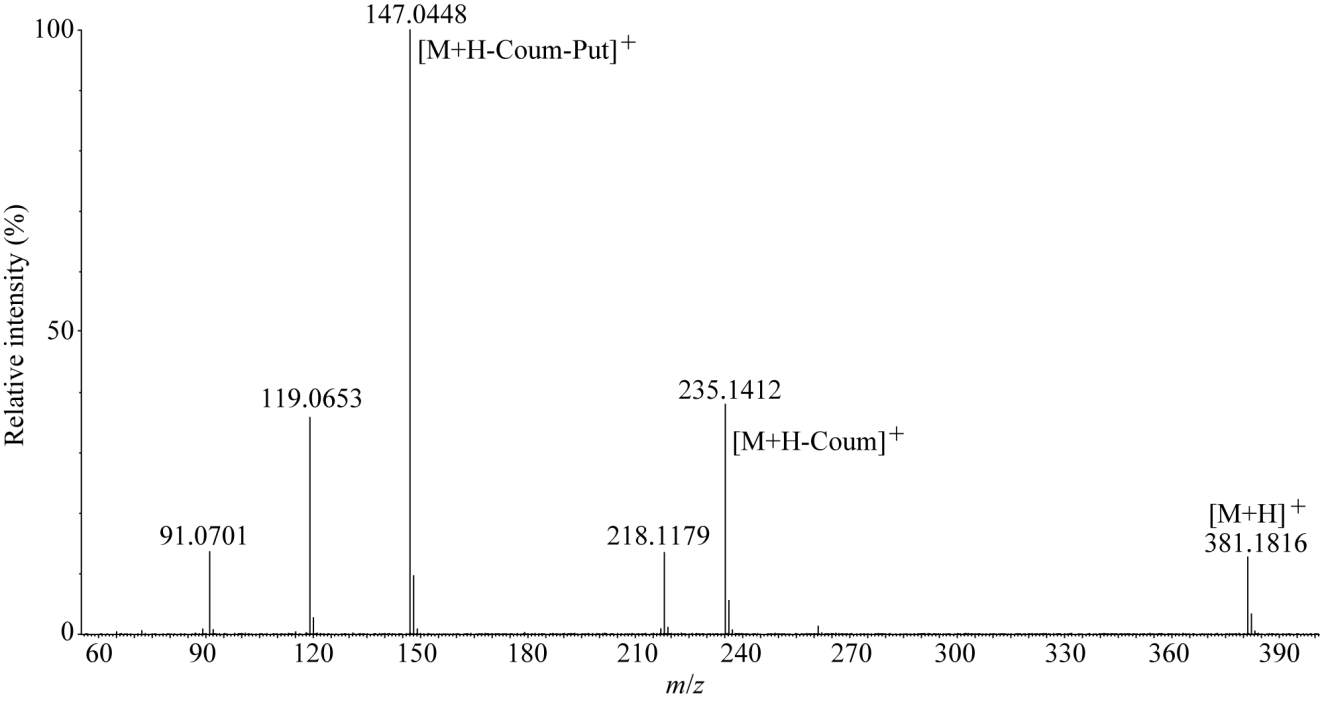


**Figure S9.** CID-MS/MS spectrum of compound 62, a putative di-*p*-coumaroylputrescine, detected from tea flowers.
